# 196 Breakthrough COVID-19 Infections Among Vaccinated Residents of VA Community Living Centers

**DOI:** 10.1017/ash.2026.10727

**Published:** 2026-06-23

**Authors:** Brandon Smith

**Affiliations:** 1 EST

## Abstract

Healthcare water management programs (WMPs) have historically emphasized the control of Legionella pneumophila, largely due to its established role in Legionnaires’ disease outbreaks. However, healthcare water systems harbor a wide range of opportunistic premise plumbing pathogens (OPPPs), including nontuberculous mycobacteria (NTM), Pseudomonas aeruginosa, Acinetobacter baumannii, Stenotrophomonas maltophilia, and Burkholderia cepacia complex. These organisms exhibit distinct ecological preferences and persistence mechanisms that vary by system type. In this study, we analyze microbiological testing data from more than 200,000 healthcare-associated water system samples collected since September 2019, comparing pathogen positivity across ice machines, general potable water, and cooling towers. Results demonstrate substantial variability in pathogen positivity by system type, with NTM showing markedly higher positivity in ice machines and potable water systems (relative to traditional indicator organisms). Legionella pneumophila positivity was highest in cooling towers but substantially lower in potable water. These findings indicate that uniform, organism-agnostic surveillance strategies are insufficient and that WMPs must be selectively designed based on system type, exposure route, and patient risk. Adopting site-specific, organism-selective monitoring frameworks may significantly improve infection prevention efforts in healthcare settings.

